# Modelling zero-truncated overdispersed antenatal health care count data of women in Bangladesh

**DOI:** 10.1371/journal.pone.0227824

**Published:** 2020-01-14

**Authors:** Zakir Hossain, Rozina Akter, Nasrin Sultana, Enamul Kabir

**Affiliations:** 1 Department of Statistics, University of Dhaka, Dhaka, Bangladesh; 2 Department of Biostatistics & Data Science, University of Kansas Medical Center, Kansas City, United States of America; 3 School of Sciences, University of Southern Queensland, Toowoomba, Australia; Victoria University, AUSTRALIA

## Abstract

Overdispersion in count data analysis is very common in many practical fields of health sciences. Ignorance of the presence of overdispersion in such data analysis may cause misleading inferences and thus lead to incorrect interpretations of the results. Researchers should account for the consequences of overdispersion and need to select the correct choice of models for the analysis of such data. In this paper, Generalized Linear Models (GLMs) are applied in modelling and analysis of antenatal care (ANC) count data extracted from the Bangladesh Demographic and Health Survey (BDHS) 2014. Pearson chi-square and different score tests are used to investigate the effect of overdispersion in the analysis. Overdispersion is found to be significant in the antenatal health care count data and so appropriate modelling is used to produce valid inferences for the regression parameters. The zero-truncated negative binomial regression (0-NBR) is found to be the best choice for analysing such data while excluding zero counts. Study findings reveal that place of residence, order of birth, exposure to mass media, wealth index and education of mother have significant impacts on the ANC status of women during pregnancy in Bangladesh.

## Introduction

In recent years, modelling of count response data is of primary interest in many fields such as public health, epidemiology and demography. Poisson regression (PR) is commonly used as the base or standard model for the analysis of count data. However, PR has a limited number of applications because of the assumption of equidispersion i.e., equality of population mean and variance. In practice, overdispersion is a common phenomenon in count data as the variance is often found to be substantially greater than the mean of counts. Thus, by ignoring the overdispersion in the analysis of count data, the analytical results produce misleading conclusions. In this case, overdispersion should be taken into consideration for modelling count data to avoid misleading inferences about the regression parameters. The negative binomial regression (NBR) is an alternative [1] to accommodate such overdispersion to the PR model. Moreover, the 0-NBR may be a further improvement for modelling count responses when there are no zero counts, for example, number of ever born children [2]; number of visits to a medically trained health care provider of women, especially those who received ANC during the whole course of pregnancy to ensure safe birth.

Maternal health care, especially during pregnancy, is a significant issue for the development of a country. Millions of women face major life-threatening complications during pregnancy in developing countries like Bangladesh [3–6]. Although pregnancy and delivery complications are very common among Bangladeshi women, very little attention has been paid by the researchers and relevant authorities to reducing these complications by ensuring adequate ANC visits during their period of pregnancy. Currently, every day approximately 830 maternal deaths occur worldwide due to pregnancy and delivery complications, and unfortunately, developing countries are responsible for 99% of these global maternal deaths [7]. Between 1990 and 2015, the maternal mortality ratio (MMR) per 100,000 live births, has decreased by about 44% worldwide [7]. However, during this time, the scenario has not improved in developing countries. It is observed that the MMR in Bangladesh has now stable in recent years as the estimated MMR is 196 maternal deaths in 2016 which is almost same as it was in 2010 [8]. One target of the sustainable development goals (SDGs) 3 is to reduce the MMR to less than 70 maternal deaths per 100,000 live births by 2030 [9], which is obviously a challenging issue.

ANC aids women to be ready for the safe delivery and to know the consequences of pregnancy and childbirth linked complications. The interventions of ANC, and particularly the number of interventions also contribute significantly to reducing maternal deaths [10] and reducing pregnancy and delivery complications [11]. It is recommended by the World Health Organization (WHO) that at least four ANC visits [12] are necessary for women during their pregnancy under normal circumstances to ensure safe pregnancy and maternal health. However, Bangladesh does not meet the target of this minimum adequate number of ANC visits [13].

There are several socio-economic and demographic factors related to the number of ANC visits of women during the pregnancy. The age at birth of women is highly significantly associated with the status of ANC visits [14]. There is a higher rate of ANC visits for women from higher socio-economic groups than those from lower socio-economic groups [15]. The impact of birth order is found to be significant and the women are less likely to receive the minimum requirement of ANC visits during the second or higher order births than their first birth [16]. Women residing in rural areas in Bangladesh are less likely to receive at least four ANC visits than women from urban areas [17]. Women’s education is also reported as a potential risk factor for standard ANC visits, antenatal morbidity and maternal health care during pregnancy [5, 6, 18, 19]. A community with lower ANC for women during pregnancy has an impact on whether women stay at home during delivery. As a result there is a high chance for such women of facing pregnancy and delivery complications [20].

It is evident from BDHS, 2014 that a significant part of Bangladeshi pregnant women does not complete the minimum standard requirement of the number of ANC visits (at least four) during the whole course of pregnancy in order to ensure the safe motherhood. More precisely, 20.7% of women visit only once, 21% visit twice and 17.4% visit three times (Table 1) i.e., in total 59.1% of Bangladeshi women visit less than four times, whereas the standard number of ANC visits is at least four [12] for ensuring the safe pregnancy and child birth. As a result there is a high likelihood for facing major life-threatening complications during the pregnancy among women who do not complete the minimum standard number of ANC visits than their counterparts. Thus, by ensuring the minimum ANC visits, women can reduce pregnancy complications and hence give safe birth. This consequently can significantly contribute for reducing maternal and child deaths in Bangladesh. It is a huge challenge to ensure that every pregnant woman does complete the minimum standard requirement of ANC visits in Bangladesh. There are very limited studies for determining the influencing factors associated with the increasing trend of ANC visits of pregnant women in Bangladesh. Therefore, in this paper, we aim to investigate the potential determinants for the adequate number of ANC visits of women receiving antenatal care from a medically trained provider in Bangladesh adopting appropriate statistical modelling using the latest secondary data on maternal health care extracted from BDHS, 2014. Overdispersion has been modelled, and tested by statistical methods and found to be significant in the data set used in this paper. For modelling such antenatal health care overdispersed count data, the overdispersed nature of the data has been completely ignored in the previous studies; however, it has been taken into account in the current analysis to avoid misleading results and conclusions.

**Table 1:** Frequency and percent distribution of ANC visiting status of pregnant women who received antenatal care from a medically trained health care provider in Bangladesh.

Number of ANC visits	Frequency	Percentage (%)
1	719	20.7
2	731	21.0
3	605	17.4
4 or higher	1423	40.9
Total	3478	100

## Materials and methods

### Source of data and sampling design

We used the nationally representative survey data from BDHS, 2014 (latest survey with information on maternal antenatal health care during pregnancy). A two-stage stratified random sampling design was used to collect the data. Bangladesh was stratified into 20 sampling strata in the survey. In the first stage, the survey used the proportional probability of sizes to the enumeration areas (EAs) and 600 EAs were selected randomly and independently in each stratum. In the survey, 207 EAs were selected from urban regions and 393 EAs from rural places. A complete list of households was then made in all of these chosen EAs. This list was used as the second stage sampling frame for the selection of households. From this listing of households, 30 households were chosen systematically from each EA with an equal probability of selection in the second stage. Finally, 18000 households were utilized in the survey; 6210 and 11790 households were in urban and rural areas respectively. Information relating to females was collected from women in the age group 15-49 who were or had been married and stayed the night before the survey date in the selected households.

### Variables included in the study

In this study, we aim to find the potential determinants for the antenatal health care count visiting status (i.e., number of visits to any ANC health care provider) of women who received ANC during their pregnancy in Bangladesh. To reflect the up-to-date scenario of antenatal health care, information was used from those women had given birth to a live baby in 5 years preceding the survey. From these figures, the data set of 6957 women was established. A number of socio-economic, demographic and community facility allied variables such as region, type of place of residence, birth order, mother’s age at birth, exposure of mass media, educational attainment, wealth index, membership of non-government organizations (NGOs) and women’s empowerment were considered as covariates. Information on all of these variables of interest were not obtained directly from the survey data. The associated covariates found in the data set were combined to compute the new variables: exposure of media, NGO membership and women’s empowerment. The variable ‘exposure of media’ was categorized using the information of women who read newspapers or magazines or listen to radio or watch television at least once per week as being exposed to media. Women are considered to be NGO members if they are associated with any one of the non-government organizations: Grameen Bank, Bangladesh Rural Advancement Committee (BRAC), Bangladesh Rural Development Board (BRDB), Association of Social Advancement(ASA), Proshika, or Mother’s Club. Women who can take the decision independently on at least one of the aspects: personal health care, purchasing main household goods, children’s health care, and independently visiting family or relatives are considered to be empowered. Removing all the missing cases in these particular variables and considering women who took ANC from any medically trained provider, finally the data set consisted of 3478 observations. The ANC count data (mean = 3.53, variance = 5.658 i.e., greater variability) of visiting status to any health care provider of Bangladeshi women during their pregnancy is given in Table 1.

### Overdispersion

Overdispersion arises when the variance of responses is higher than the mean in a PR model. This is a common phenomenon in a real count data set. The violation of distributional assumptions may also cause overdispersion. Therefore, to avoid misleading inferences and results, overdispersion should be taken into consideration for analyzing the antenatal health care count data of women during pregnancy in Bangladesh. In order to detect the presence of overdispersion in a data set, the value of the Pearson residual chi-square (*χ*^2^) statistic [1, 21] divided by the corresponding degrees of freedom (*df*) is used. This value is known as the *dispersion*. If the value of *dispersion* is greater than 1, then the model is overdispersed, the value 1 is for equidispersed and less than 1 for underdispersed model. The Pearson *χ*^2^-statistic is defined as

$$\begin{array}{c} \text{Pearson}\;\chi^{2}=\sum_{i=1}^{n}\frac{{\left(y_{i}-\mu_{i}\right)}^{2}}{V(\mu_{i})}, \end{array}$$

where *y*_*i*_ and *μ*_*i*_ represent the observed and expected counts respectively. Moreover, the variance function *V* is equal to *μ*_*i*_ and $\mu_{i}+k\mu_{i}^{{}^{2}}$ for the PR and NBR models respectively, where *k* is the dispersion parameter.

Overdispersion in a real count data set is natural. A natural question then may arise whether the amount of overdispersion is statistically significant or not and should be taken into consideration in the analysis of such data. However, statistical tests need to be conducted and this can be done by using the score test. Three versions of the score test statistic are defined by Dean and Lawless (1989) [22]:

$$\begin{array}{c} z_{i}=\frac{\left(y_{i}-\mu_{i}^{2}\right)-y_{i}}{\sqrt{2}\mu_{i}}, \end{array}$$

Cameron and Trivedi (1990) [23]:

$$\begin{array}{c} z_{i}=\frac{\left(y_{i}-\mu_{i}^{2}\right)-y_{i}}{\mu_{i}} \end{array}$$

and Winkelmann (2008) [24]:

$$\begin{array}{c} z_{i}=\frac{\left(y_{i}-\mu_{i}^{2}\right)-y_{i}}{2\mu_{i}}. \end{array}$$

### Models

We consider three regression models: Poisson, negative binomial (NB) and zero-truncated negative binomial (0-NB) in the context of the GLMs framework [25, 26] for modelling and analysis of antenatal health care count data in Bangladesh. The modelling and analysis of count response data may start with PR regression as the base or standard model. However, count data are often found to be overdispersed in the practical field. This was the case for the data set considered in this study. Therefore, one may consider the NBR as an alternative for further improvement of the modelling and data analysis. In case of the NBR, the multiplicative random effect *U* is used to model the unobserved variability in the data set. Let *Y* be the count response variable and then the conditional distribution of *Y* given an unobserved random variable *U* is Poisson with mean and variance equal to λ*u* i.e., *Y*|*U* ∼ Poisson (λ*u*). For computational simplicity, the random effect *U* is assumed to follow the gamma distribution with shape parameter *δ* and scale parameter *δ*^−1^ i.e., *U* ∼ Gamma $\left(\delta ,\frac{1}{\delta }\right)$. Thus the unconditional marginal distribution of *Y* is the NB distribution with mean *E*(*Y*) = *μ* and variance *V*(*Y*) = *μ* + *κμ*^2^ where *μ* = λ > 0 and the overdispersion parameter *κ* = *δ*^−1^. It follows that the variance is greater than the mean as *κ* > 0 and consequently the overdispersion in the data is accounted for the NBR in contrast to the PR.

It can also be shown that the Poisson and NB distributions belong to the exponential family. In GLMs using the link function *g*, the mean function of responses *g*(*μ*) is modelled in terms of a set of covariates and regression parameters. In order to construct GLMs for Poisson and NB responses the natural logarithm may be considered as the link function. It permits the comparison of NB with *κ* = 0 directly with the PR model [1]. Let ***x***_*i*_ = (*x*_*i*1_, …, *x*_*ip*_)^⊤^ be the *p* × 1 vector of covariates and ***β*** = (*β*_1_, …, *β*_*p*_)^⊤^ represents the vector of regression parameters of order *p* × 1. Then the form of a GLM may be written as $\text{ln}\left(\mu \right)=x_{i}^{⊤}\beta$. In the case of the NBR model, we estimate ***θ*** = (***β***, *κ*)^⊤^ by maximizing the log-likelihood [1] function *l*(***θ***, ***y***), given by

$$\begin{array}{c} l_{NB}\left(\theta ;y\right)=\sum_{i=1}^{n}[y_{i}\ln(\frac{\kappa \mu_{i}}{1+\kappa \mu_{i}})-\frac{1}{\kappa }\ln\left(1+\kappa \mu_{i}\right)+\ln(\frac{(y_{i}+\kappa^{-1}-1)!}{y_{i}!\left(\kappa^{-1}-1\right)!})]. \end{array}$$

The response variable, the number of visits to a medically trained health care provider of women who received antenatal care during the pregnancy, excludes zero counts and one may then consider 0-NB distribution. The probability of zero counts, (1 + *κμ*)^*κ*^, is subtracted from the total probability 1 and the required modifications are performed to develop the corresponding theory for 0-NBR. The log-likelihood [1] can also be given by

$$\begin{array}{c} l_{0-NB}\left(\theta ;y|y>0\right)=\sum_{i=1}^{n}[l_{NB}\left(\theta ;y\right)-\ln\{1-{\left(1+\kappa e^{x_{i}^{⊤}\beta }\right)}^{-\frac{1}{\kappa }}\}]. \end{array}$$

The model selection criterion, Akaike information criterion (AIC), is considered to select the best choice of models with smallest AIC value [27] for the data analysis and is given by

$$\begin{array}{c} AIC=-2l(\hat{\theta };y)+2p, \end{array}$$

where $\hat{\theta }$ is the vector of estimated model parameters and *p* is the number of parameters. For interpretation of results it is convenient to use the incidence rate ratio (IRR) in order to investigate the effect of covariates on the count response variable rather than the regression coefficients. The estimated *IRR* for the individual covariate *x*_*j*_ is defined as

$$\begin{array}{c} IRR_{j}=e^{{\hat{\beta }}_{j}}, \end{array}$$

where ${\hat{\beta }}_{j}$ is the *j*-th estimated regression coefficient for *j* = *i*, …, *p*.

### Ethics statement

This article does not contain any studies with human participants performed by any of the authors. The Bangladesh Demographic and Health Surveys (BDHS) were approved by ICF Macro Institutional Review Board and the National Research Ethics Committee of the Bangladesh Medical Research Council. A written consent about the survey was given to participants before interview. All identification of the respondents was dis-identified before publishing data. The secondary data are used in the current study and freely available on the DHS website (https://dhsprogram.com/data/available-datasets.cfm).

## Results and discussion

In this study, we first investigated whether overdispersion was present or not in the antenatal health care count data of Bangladeshi pregnant women. In order to do this, we fit PR model and then computed the *dispersion* value by using Pearson *χ*^2^-statistic. From Table 2, it can be seen that the Pearson dispersion value is 1.394 which clearly indicates the presence of overdispersion in the data and moreover the PR model is overdispersed. We then conducted the score test with PR model to examine the presence of overdispersion whether this is statistically significant or not for the violation of model assumptions. The results are given in the right panel of Table 2.

**Table 2:** Detection of overdispersion based on Pearson residual *χ*^2^-statistic for different models with AIC values and score tests of significance of overdispersion to antenatal health care count data in Bangladesh.

Detection and Model Selection			Test (PR model)			
Model	*Dispersion*	AIC	Method	z-score	SE	*p*-value
PR	1.394	4.228	Dean and Lawless	0.276	0.029	<0.001
NBR	1.056	4.178	Winkelmann	0.193	0.020	<0.001
0-NBR	**1.003**	**4.024**	Cameron and Trivedi	0.385	0.041	<0.001

From *p*-values (*p*<0.001) in Table 2, it is evident that overdispersion is significant and the PR model is observed to be overdispersed. It follows that one should consider the alternatives for modelling and analysis of such overdispersed antenatal health care count response data. We then fit NBR and calculate the Pearson dispersion value. From Table 2, it can be seen that this value is 1.056 which is close to 1 and it seems to be that overdispersion has been captured well by the NBR model. However, the count response variable (number of visits to a medically trained provider among women who received antenatal health care) excludes zero visits in this study. Therefore, we consider 0-NBR model as a further improvement for modelling and analysis of the antenatal heath care count data. We then again compute the value of *dispersion* and this is 1.003. This indicates that the overdispersion in the data set has been modelled and captured very well by 0-NBR as the *dispersion* value is very close to 1. Finally, we choose the 0-NBR model for the data analysis (smallest AIC = 4.024) in order to investigate the impact of potential determinants on antenatal health care of women during their pregnancy in Bangladesh. The summary results obtained from fitting this model are presented in Table 3.

**Table 3:** Impact of socio-economic and demographic determinants on antenatal health care of women during pregnancy in Bangladesh along with parameter estimates, standard errors (SE), *p*-values and IRRs obtained from fitting 0-NBR model.

Covariate	Estimate	SE	*p*-value	IRR
Intercept	0.657	0.073	<0.001	–
**Division**				
Barisal	-0.043	0.051	0.400	0.96
Chittagong	-0.069	0.043	0.112	0.93
Dhaka	–	–	–	–
Khulna	0.133	0.046	0.004	1.14
Rajshahi	-0.008	0.048	0.874	0.99
Rangpur	0.200	0.047	<0.001	1.22
Sylhet	-0.069	0.050	0.166	0.93
**Place of residence**				
Rural	–	–	–	–
Urban	0.176	0.030	<0.001	1.19
**Birth order**				
1	0.032	0.035	0.356	1.03
2–3	–	–	–	–
4 or higher	-0.141	0.053	0.008	0.87
**Mother’s age at birth (years)**				
<20	-0.070	0.036	0.050	0.93
20–35	–	–	–	–
<35	-0.019	0.086	0.823	0.98
**Exposure of media**				
Non–exposed	–	–	–	–
Exposed	0.108	0.037	0.004	1.11
**Educational attainment**				
No education	–	–	–	–
Primary	0.119	0.059	0.043	1.13
Secondary	0.249	0.057	<0.001	1.28
Higher	0.476	0.065	<0.001	1.61
**Wealth index**				
Poor	0.003	0.043	0.940	1.00
middle	–	–	–	–
Rich	0.110	0.039	<0.001	1.12
**NGO membership**				
No	–	–	–	–
Yes	0.033	0.034	0.320	1.03
**Women empowerment**				
No	–	–	–	–
Yes	0.033	0.032	0.295	1.03

It is observed from IRR and *p*-values that the average number of ANC visits of women during pregnancy from Khulna and Rangpur divisions are 1.14 times and 1.22 times more than women who live in Dhaka division and are statistically significant. Although the average number of ANC visits of pregnant women from Barisal, Chittagong, Rajshahi and Sylhet are respectively 4%, 7%, 1% and 7% lower than women in the Dhaka region but they are not statistically significant. The place of residence of women has a highly statistically significant (*p*<0.001) effect on the number of antenatal health care visits of women during the course of pregnancy. The average antenatal care visits of urban women during pregnancy are 1.19 times more than those of rural women.

The birth order of women is found to be significant in relation to receiving antenatal care from a medically trained provider during the pregnancy. More specifically, the average number of antenatal health care visits is 13% lower during the pregnancy of fourth and later birth (IRR = 0.87) than the duration of the pregnancy of the second and third birth. The mean number of antenatal health care visits of women during pregnancy is 7% (*p* = 0.050) less for women aged 20 years or below compared to women of age group 20-35 years at the time of delivering their baby. Exposure of media (*p* = 0.004) is found to be statistically significant for receiving antenatal health care during the duration of their pregnancy. The average number of ANC visits is 11% higher in women who are exposed to media compared with their counterparts (IRR = 1.11). From the reported values of IRRs for different levels of education of women, it is clear that the average number of ANC visits increases significantly (primary: *p* = 0.043; secondary and higher: *p*<0.001) with the increasing level of their education. Women who completed primary and secondary levels of education are 1.13 times and 1.28 times higher respectively to receive antenatal care from a medically trained health care provider during the course of pregnancy than women who have no education. On average, the number of ANC visits to a health care provider of highly educated women is 1.61 times higher compared with uneducated women.

It may also be clearly seen that the average number of antenatal care visits of women during pregnancy from a rich family (*p*<0.001) is 12% more than that of women who belong to middle class families. Furthermore, the average number of antenatal health care visits for the women belonging to the NGO membership group and contributing in taking important decisions 3% higher than for women who are not members of NGOs or not involving in taking any important decision, though both of them are statistically insignificant.

## Conclusion

The study results reveal that one should account for the issue of overdispersion in a real count data set to assess precisely the significance of regression parameters. It is also necessary to test whether the overdispersion is significant or not and then one may select the suitable model for the analysis of count response data. We used the latest survey data (BDHS, 2014) in order to investigate the influencing factors associated with antenatal health care of women during the course of pregnancy in Bangladesh. Due to overdispersion in the data, the zero-truncated negative binomial model was chosen for its suitability to estimate the average number of antenatal care visits of Bangladeshi women. The findings from the analysis show that region or division, place of residence, birth order, exposure of media, educational status of women and wealth index have significant effect on the number of antenatal visits to a medically trained health care provider. The significant factors, place of residence, education, wealth index and exposure of media have positive effects while the birth order has negative effects on the antenatal health care visits of Bangladeshi women. More precisely, women from urban areas, belonging to rich families, with a higher level of education, and who are also exposed to media receive more antenatal care by visiting medically trained health providers. Moreover, women at the time of fourth and later birth receive less antenatal care compared to the second and third birth pregnancy in Bangladesh.

Based on the findings in this study, awareness should be created among the women who give birth their first child at the age of below or equal to 20 years so that they can receive more antenatal health care during pregnancy. Moreover, effective attention should be given to motivate women for receiving more antenatal care irrespective of their birth order for a safe birth. Education of women needs to be given top priority and access to media of women also should be facilitated for better antenatal care in Bangladesh. Moreover, basic appropriate maternal health care services must be ensured in rural areas and among the women belonging to poor families in order to reduce the risk of pregnancy complications, and maternal and child morbidity and mortality in Bangladesh.
